# Understanding the Role of Psychiatric Principles in Patient Care: An Important Goal of the Medical Student Clerkship in Psychiatry

**DOI:** 10.3389/fpubh.2016.00030

**Published:** 2016-02-29

**Authors:** Leo Sher, Antonia S. New

**Affiliations:** ^1^James J. Peters Veterans’ Administration Medical Center and Icahn School of Medicine at Mount Sinai, New York, NY, USA

**Keywords:** psychiatry, medical student, education, clerkship, mentorship, stigma, suicide

## Medical Student Clerkship in Psychiatry

The significance of the psychiatry clerkship in medical student education is broadly and internationally recognized (1). In the United States, all 126 allopathic medical schools oblige their students to complete a clinical clerkship in psychiatry (2). In medical schools in the United States, the length of this clerkship is 4–6 weeks (3–5). According to the “A Position Statement on the Length of the Psychiatry Clerkship” published in 2006 by the Association of Directors of Medical Student Education in Psychiatry, “the psychiatry clerkship must be at least 6 weeks in length or longer” (6). In some non-U.S. Medical Schools, the duration of the psychiatry clerkship is 8–10 weeks (7, 8).

The medical student psychiatry clerkship has taken an increased importance as the clinical practice of medicine evolves. Clinical training in psychiatry is a necessary part of a general medical education (9). Medical educators suggest that “any physician … must be able to diagnose, treat, manage, and refer patients with psychiatric disorders” (10). However, most physicians receive little training in interacting with patients with major mental illness and can feel uncomfortable or ineffective communicating with them, even about physical complaints (11–13).

Students should understand that the knowledge of psychiatric principles is important for non-psychiatric physicians. Studies have demonstrated that about 20–40% of patients in primary care settings have a diagnosable psychiatric illness (14–18). The annual prevalence of all psychiatric disorders, including substance use disorder, is about 30% in the United States (19). The lifetime prevalence of any psychiatric disorder in the United States is >45% (20). Despite this, there is underrecognition of psychiatric conditions in the primary care setting (17, 21, 22).

To understand psychiatric principles in patient care, students should obtain a foundation of knowledge concerning the psychological and humanistic aspects of the practice of medicine based on the study of the behavioral sciences and clinical psychiatry; learn interpersonal skills related to the management of patients with medical and/or psychiatric diseases; and follow attitudes and values that enhance the professional roles of physicians (23, 24).

Suicide and intentional self-harm are major health-care issues. A research report suggests that patients hospitalized in a general hospital have an eight times higher risk of committing suicide compared to the general population (25). Many physicians who work with medical or surgical patients do not have adequate training in suicide assessment techniques and treatment approaches to suicidal patients (26, 27). Patients who commit suicide in general (i.e., non-psychiatric) hospitals have a different profile than patients in psychiatric hospitals or those who commit suicide in the community (28). For example, general hospital patients who commit suicide are less likely to have a known history of psychiatric illness or suicidal behavior (29). Therefore, knowledge in suicide and intentional self-harm ­prevention is important for all physicians and should be one of the focuses of the medical student clerkship in psychiatry (30–32).

## Stigma of Psychiatric Illness and Medical Student Education

Mental illness is often related to social stigmatization, discrimination, and prejudice and people with mental illness are often perceived as dangerous, unpredictable, and aggressive (33–38). This stigma discourages involvement in health care and results in mistreatment of patients with psychiatric disorders. Education and contact with persons with mental illness are associated with reduced stigma. It is important to explain to the students that psychiatric disorders are diseases of the brain. They are medical conditions, such as hypertension, diabetes, hepatitis, and nephritis, and psychiatric disorders, such as mania, melancholia, and hysteria, have been included in classifications of diseases since the time of Hippocrates, and for over 2000 years were treated by physicians with the same kind of remedies, pharmaceuticals, and attempts to adjust humoral imbalance as they used for other more obviously medical conditions (39, 40). The idea that insanity was fundamentally different from other illnesses, that it was a disease of the mind rather than the body, was only developed toward the end of the eighteenth century. However, by the middle of the nineteenth century, it was commonly accepted that the superintendent of any properly run “lunatic asylum” should be a physician.

It is important for students to understand that psychiatric disorders should be assessed and successfully treated from the perspective of the biopsychosocial model (41–43). Dr. George L. Engel, the founder of the biopsychosocial model suggested that “all three levels, biological, psychological, and social, must be taken into account in every health care task” (41, 42). Engel criticized a cold, impersonal, technical, and biomedically oriented style of clinical practice. Biological, psychological (thoughts, emotions, and behaviors), and social (including cultural) factors play a significant role in the pathophysiology of psychiatric disorders. No single illness, patient, or condition can be reduced to any one aspect.

## The Impact of the Psychiatry Clerkship

A recent study examined the literature on the impact of the psychiatry clerkship to determine the effect on attitudes toward psychiatry and to psychiatry as a career (44). Twenty-six studies from 19 countries were evaluated. The authors found that the psychiatry clerkship has a positive impact on students’ attitudes toward psychiatry, but does not improve interest in psychiatry as a career option. This study suggests that charismatic teachers, good mentorship, and stigma reduction improve students’ attitude toward psychiatry. It is important, however, to note that there may be significant cross-cultural differences with regard to the medical student education and stigma associated with psychiatric illness.

A British study examined determinants of student attitudes to psychiatry and intentions to pursue psychiatry as a career (7). Three hundred seventy-nine medical students completed questionnaires at the beginning and end of an 8-week psychiatry clerkship. The authors found that student attitudes to psychiatry improved and intentions to pursue psychiatry as a career increased during the clerkship. These changes were predicted by specific experiences during the clerkship, such as receiving encouragement from supervisors, seeing patients who respond well to treatment, and having direct involvement in patient care. A recent survey of final year medical students at 46 medical schools in 20 countries showed that female gender, experience of mental or physical illness, media portrayal of doctors, and positive attitudes to psychiatry, but not personality factors, were associated with choosing psychiatry (45). The authors of the study also observed that during medical school, experience of psychiatric enrichment activities (such as psychiatry clubs), experience of acutely unwell patients and perceived clinical responsibility were all associated with choice of psychiatry. A recent research paper suggests that the integration of strategies to overcome stigma, both toward people with mental illness and the mental health profession, into preclinical teaching may provide students with skills to better prepare them for the psychiatry clerkship and could assist in improving their attitudes toward psychiatry (46).

In summary, studies have shown that experience during a psychiatry clerkship affects the attitude of medical students toward psychiatry. It is important to note that relatively few studies of this subject have been conducted.

## Some Important Considerations Related to the Psychiatry Clerkship

Many factors affect the quality of the psychiatry clerkship. Some recommendations are listed below (1, 3–5, 23, 24, 46, 47):

• Supervision during the psychiatry clerkship is very important. Every student of medicine should be accompanied by a supervising physician.
• If students work with mostly very severe cases, such as cases of disorganized schizophrenia, the clerkship in psychiatry could have negative impact on attitude toward psychiatry. The educators should try to offer a complete and correct image of psychiatry.
• The psychiatry clerkship should include both inpatient and outpatient experience. Students should also participate in clinical care in community mental health centers.
• Students should take calls with psychiatry residents.
• Students should be taught psychiatric interviewing techniques.
• Students should be taught that a psychotherapeutic approach not only a psychopharmacological treatment is important in the management of psychiatric patients. The best outcomes may be provided for many patients and for many psychiatric disorders by combining psychotherapy with psychopharmacology.
• Students should be informed about suicide medical malpractice issues. Students should know that suicide malpractice lawsuits can be filed against both psychiatrists and non-psychiatrists.

## Conclusion

A positive education experience increases the probability of a positive student attitude toward psychiatry. There are two major contributing factors to the positive experience:

(a) Enthusiastic, charismatic mentors. Mentors’ enthusiasm is recognized as one of the most essential and desirable qualities and characteristics of effective faculty members. It plays a central role in holding students’ attention, generating students’ interest, and developing students’ positive attitudes toward learning psychiatry; and
(b) Direct involvement in the patient care. Students should also participate in activities, such as rounds, group therapy, and case management. There should be an emphasis on positive outcomes and recovery for persons with psychiatric disorders. Students should understand that patients with psychiatric conditions can be successfully treated.

In order to assure adequate care for the mentally ill, it should be a fundamental aim of medical education to promote positive attitudes toward the mentally ill and psychiatry.

## Author Contributions

LS has done a literature search and worked on the manuscript. AN has worked on the manuscript.

## Conflict of Interest Statement

The authors declare that the research was conducted in the absence of any commercial or financial relationships that could be construed as a potential conflict of interest.
